# Ritual Slaughter and Supranational Jurisprudence: A European Perspective

**DOI:** 10.3390/ani15121756

**Published:** 2025-06-14

**Authors:** Michela Maria Dimuccio, Pasquale De Marzo, Virginia Conforti, Francesco Emanuele Celentano, Giancarlo Bozzo

**Affiliations:** Department of Veterinary Medicine, University of Bari, Strada Provinciale Per Casamassima Km 3, 70010 Valenzano, Italy; michela.dimuccio@uniba.it (M.M.D.); pasquale.demarzo@uniba.it (P.D.M.); v.conforti@studenti.uniba.it (V.C.); giancarlo.bozzo@uniba.it (G.B.)

**Keywords:** ritual slaughter, animal welfare, religious freedom, European Union law, stunning methods

## Abstract

Ritual slaughter—understood as the killing of animals without prior stunning for religious purposes—constitutes a legally and ethically intricate domain, situated at the intersection of animal welfare, freedom of religion, public health, and consumer protection. This review offers a critical examination of the influence exerted by international and supranational jurisprudence—most notably the case law of the Court of Justice of the European Union—on the regulatory landscape governing ritual slaughter. While the right to religious freedom enjoys robust protection under European constitutional and human rights frameworks, recent judicial decisions have affirmed the legitimacy of national legislative measures mandating pre-slaughter stunning, insofar as such measures pursue objectives of animal welfare and transparency in the public interest. Particular attention is devoted to seminal rulings originating in Belgium and within the broader EU context, with a focus on the application of the principle of proportionality as a legal mechanism for balancing colliding fundamental rights. The analysis further engages with the scientific and ethical discourse surrounding animal suffering and the legal obligations tied to consumer information and labeling. Taken together, these developments reveal an emergent trajectory within EU law toward the progressive tightening of regulatory standards governing ritual slaughter, shaped by an evolving jurisprudential understanding of animal welfare imperatives.

## Simple Summary

Ritual slaughter, as observed in Jewish and Islamic religious traditions, involves the killing of animals without prior stunning, in strict adherence to doctrinal mandates. This practice has become the focal point of ongoing legal, ethical, and animal welfare debates—particularly within the European Union, where the legal recognition of animals as sentient beings imposes heightened regulatory scrutiny. This review undertakes a critical analysis of the evolving jurisprudence of European and international courts with regard to the normative framework governing ritual slaughter. It delineates the inherent legal tensions between the fundamental right to religious freedom and the state’s duty to uphold animal welfare standards. Additionally, it examines the implications of these legal developments for food labeling practices and consumer transparency. The discussion underscores the increasing necessity of reconciling constitutionally protected cultural and religious expressions with contemporary ethical imperatives and scientifically grounded welfare norms.

## 1. Introduction

The term ritual slaughter refers to a specific method of animal killing practiced within Islamic and Jewish religious traditions. Unlike conventional methods, ritual slaughter is characterized by the absence of prior stunning. Both approaches mark the initial phase in the transformation of a live animal into meat intended for human consumption [1]. Beyond its strictly religious dimensions—and the associated right to religious freedom—this practice raises multifaceted issues, encompassing public health, economic interests, legal regulation, and, most significantly, animal welfare, particularly with regard to the suffering endured by animals in their final moments [2].

The subject remains at the center of deliberation by both legislative and judicial institutions within the European framework, as they seek to navigate the delicate balance between safeguarding animal welfare and ensuring the free exercise of religious beliefs. The increasing societal recognition of animals as sentient beings has led to what may be described as a Global Animal Law model, synthesizing ethical, moral, and legal approaches across jurisdictions [3].

An additional point of not insignificant relevance arises in the context of consumer rights: individuals must be adequately informed—primarily through appropriate labeling—of whether the meat they consume originates from animals subjected to ritual slaughter [4].

Within ritual slaughter practices, the hindquarters of animals—though ritually slaughtered—are frequently diverted to the conventional meat market without any indication as to the method of slaughter. This constitutes a legislative lacuna that deprives consumers of the right to make informed choices, thus undermining the principle of transparency.

Where the legal expression “balancing of conflicting interests” is invoked, it presupposes the existence of multiple legally protected interests. In the case at hand, the supranational legal orders of the European Union (EU), the Council of Europe (CoE), and the Court of Justice of the European Union (CJEU) are compelled to weigh religious freedom against public morality, animal protection, consumer rights, and the right to health. The complexity of this balancing exercise is such that “the compression of certain fundamental values and rights may be experienced by those who are the bearers of the relevant demand as excessive or unjust” [5].

A democratic community of states, founded upon the values of pluralism and diversity, is duty-bound to guarantee the right of every European citizen—and by extension, non-European individuals residing in or transiting through the territory—to freely exercise their religion, inclusive of rites and precepts [6,7].

In this context, the principle of proportionality assumes a central role in judicial reasoning. The judge is tasked with the nuanced responsibility of safeguarding one fundamental right, while simultaneously ensuring that others are not unduly diminished. In the specific instance of slaughter, animal welfare—especially in the pre-slaughter phase—must be weighed against public health considerations relating to the hygienic quality of meat.

For Islamic and Jewish communities, the exercise of religious belief is manifested, inter alia, through ritual slaughter, imbued with profound sacrificial and symbolic meaning. For this reason, the study of stress levels in animals slaughtered according to ritual prescriptions has involved comparative assessments with conventional methods, with the aim of evaluating both welfare outcomes and meat quality [4].

Scientific research has revealed that elevated concentrations of cortisone and cortisol metabolites in plasma, feces, urine, saliva, and milk can serve as effective indicators of stress [8].

The notion of animal welfare is generally understood to denote a sustained condition reflective of the animal’s subjective experience, evaluated on behavioral, postural, and physiological grounds [8]. However, these indicators remain subject to scientific scrutiny and debate as to “their validity as well as the reliability of the chosen well-being indicators” [9]. Particular attention must be devoted to critical procedural elements: the moment of neck incision, the stress experienced during exsanguination, the duration of bleeding and the time required to achieve loss of consciousness [10,11,12].

In the context of ritual slaughter, the process begins with jugulation absent prior stunning—a procedure that is permissible, with reversible stunning, only within certain Islamic interpretations. Some scholars assert that exsanguination induces an immediate cessation of cerebral blood flow, thereby causing instantaneous loss of consciousness and death [13,14]. Others, however, contend that full loss of consciousness may be delayed by over a minute following the initiation of exsanguination [15,16,17].

Such scientific discord underscores the inherent difficulty in establishing a universally accepted standard of “animal welfare,” particularly where ethical, cultural, and legal paradigms intersect. Furthermore, the elevated stress parameters observed during ritual slaughter may adversely affect both animal welfare and the quality of the meat produced.

With regard to normative provisions, Article 4 of Regulation (EC) No 1099/2009 mandates that, as a general rule, animals must be stunned prior to slaughter in order to induce a loss of consciousness and sensibility. However, a derogation is provided for ritual slaughter: under Article 4, such procedures may be conducted without stunning, provided they are carried out in authorized slaughterhouses and subject to rigorous supervision to ensure that animals are insensible and unconscious before being released from restraint systems. Only upon fulfillment of these preconditions may the slaughtering and meat processing operations proceed in accordance with the regulation [18].

The objective of this review is to critically examine how selected decisions of European and international courts have engaged with the normative and ethical complexities surrounding ritual slaughter practices. Anchored in a jurisprudential perspective, the analysis focuses on the legal methodologies employed to reconcile the protection of core fundamental rights—such as the freedom of religion—with contemporary scientific and ethical advances in the domain of animal welfare. By mapping out key judicial developments, the study aims to elucidate the evolving role of legal systems in regulating religious slaughter within the broader European legal landscape.

## 2. Methods

This narrative review was conducted through a structured search of legal databases and scholarly repositories to identify key sources addressing ritual slaughter, animal welfare, and the protection of religious freedom within European and international legal frameworks. The search was performed between January and April 2025 using platforms such as EUR-Lex, HUDOC, Scopus, Google Scholar, and PubMed in their current 2025 version. The queries included combinations of the terms “ritual slaughter”, “animal welfare”, “religious freedom”, “EU law,” “Halal”, “Kosher”, “stunning”, and “proportionality principle”. A total of 52 documents were initially retrieved, including judicial rulings, EU and international legal texts, peer-reviewed articles, and legal commentaries. Of these, 19 were excluded after title and abstract screening due to lack of relevance to the legal governance of ritual slaughter or insufficient academic rigor (e.g., non-peer-reviewed materials). An additional 6 documents were excluded during full-text review for reasons such as duplication or failure to address the interaction between religious rights and animal welfare. The final sample comprised 27 sources, of which 5 were landmark court decisions (mainly from the CJEU and ECtHR) and 22 were scholarly or institutional contributions in English or Italian. Sources were selected based on (1) direct relevance to the regulation of ritual slaughter within the EU or Council of Europe; (2) citation significance or institutional authority; and (3) legal or scientific credibility. The selected materials were analyzed to map the evolving legal interpretation of ritual slaughter, especially concerning the balancing of fundamental rights (e.g., religious freedom) with public interest objectives such as animal welfare and consumer protection.

## 3. Animal Welfare and Ritual Slaughter in European Union Law

The concept of animal welfare—which underpins the present analysis—first emerged within the framework of EU law with the Treaty of Amsterdam in 1997, before being formally enshrined as a foundational principle in the Treaty of Lisbon, which entered into force on 1 December 2009 [19,20]. The notion of “animal welfare” is inherently multidisciplinary; prior to acquiring legal force, it had already been the subject of extensive philosophical and bioethical inquiry. Article 13 of the Treaty on the Functioning of the European Union (TFEU) provides that “the Union and the Member States shall pay full regard to the welfare requirements of animals as sentient beings” [20]. However, this provision is drafted in broad and interpretative terms, rendering its scope legally indeterminate.

The same article continues by stipulating that such regard for animal welfare shall be exercised “while respecting the legislative or administrative provisions and customs of the Member States relating, in particular, to religious rites, cultural traditions and regional heritage” [20]. This formulation encapsulates the essence of the balancing exercise central to this field. The Court of Justice of the European Union (CJEU) has progressively construed “animal welfare” as a value of public interest, thereby expanding its protective scope—at times at the expense of competing rights and interests [20].

In the Zuchtvieh Export judgment of 23 April 2015 [21], the Court referred to the concept of “animal welfare” in the context of live animal transport, invoking Regulation (EC) No 1/2005 [22]. The ruling endorsed an expansive interpretation of “animal welfare,” extending its applicability to the movement of animals both from EU territory to third countries and vice versa [22].

The Brouwer judgment further developed this interpretation [23]. There, the Court revisited the prohibition against tethering calves for breeding purposes. The judgment extended the protective scope of “welfare” not only to calves “confined for rearing and fattening” but also to those tethered solely for milk production [23]. The jurisprudential elaboration of “animal welfare” has thus evolved in a case-by-case manner, grounded in the factual matrix of each dispute.

For instance, in Pfotenhilfe Ungarn, the Court contributed further to the conceptual framework of animal welfare in the context of transporting stray dogs across EU Member States [24]. The German Federal Administrative Court had requested a preliminary ruling to assess the conduct of a non-profit association engaged in the transportation of animals for the purpose of adoption. The issue arose as to whether the association’s request for reimbursement could be construed as a profit-generating activity. The CJEU was thereby tasked with determining whether such operations constituted an “economic activity” within the meaning of EU law [24].

The classification of the activity as economic rendered Regulation (EC) No. 1/2005 applicable, thereby invoking the corresponding “animal welfare” standards pertaining to transport [22]. The Court acknowledged that its jurisprudence on animal welfare remains in a state of ongoing development—indeed, some findings appear to be in tension with prior rulings. For example, in an earlier decision, the same Court had held that an individual’s request for reimbursement did not qualify as an economic activity [25].

In another notable case, in terms of the European Federation for Cosmetic Ingredients, Advocate General Michal Bobek emphasized and reaffirmed the objective of “the protection of animal welfare,” while simultaneously linking it to the safeguarding of human health. He further underscored the relative nature of this principle in relation to the overarching interest in human well-being [26].

The evolving legal and conceptual framework surrounding “animal welfare” plays a determinative role in the jurisprudence concerning ritual slaughter. A synthesis of the most relevant judgments is provided in Table 1, which offers a concise overview of how European courts have adjudicated the tension between the protection of religious freedom and the imperative of animal protection (Table 1).

Ritual slaughter is defined as the practice of killing animals without prior stunning, in observance of the religious prescriptions of the Muslim and Jewish faiths. While the respective rituals of these traditions differ in certain procedural aspects, both require the animal to remain conscious until death, which is induced through jugulation. Although often viewed through a religious and legal lens, the issue is undeniably rooted in a broader cultural and historical context. As gruesome as the act may appear, it is worth recalling that both the Qur’an and Jewish law contain detailed injunctions on how to slaughter animals using techniques designed to limit suffering—codified centuries before the development of mechanical stunning methods [26].

In 2018, the Court of Justice of the European Union (CJEU) was called upon to interpret the provisions of Regulation (EC) No 1099/2009 in the context of ritual slaughter. The matter arose in Liga van Moskeeën en Islamitische Organisaties Provincie Antwerpen, VZW and Others v. Vlaams Gewest (29 May 2018) [27]. The legal question was whether ritual slaughter without prior stunning could be lawfully restricted to authorized slaughterhouses and, if so, whether such a requirement infringed upon the free exercise of the Islamic faith. The concern was that requiring slaughter to occur exclusively in approved facilities would hinder the availability of halal meat during key religious observances, such as “the feast day,” thereby impeding religious practice.

Additionally, the restriction raised economic concerns: the limited number of authorized facilities in the region necessitated the transportation of ritual meat from other areas, imposing significant logistical and financial burdens on the faithful [27]. Nevertheless, the Court held that such logistical and economic consequences did not amount to a direct violation of religious freedom. For a breach of this fundamental right to occur, the interference must be direct—for instance, an outright prohibition on the exercise of religious rites—and not merely incidental, such as increased cost or logistical inconvenience.

Thus, the application of Regulation (EC) No 1099/2009 was not deemed a per se infringement of the right to freedom of religion. Another contentious point involved the interpretation of Article 13 TFEU, which simultaneously upholds animal welfare while mandating respect for “the laws and regulations of the Member States relating to religious rites, cultural traditions and cultural heritage” [18,20]. The Court concluded that the measure in question did not contravene this provision, since the regulation did not explicitly prohibit religious rites, but rather sought to safeguard animal welfare in a proportionate and non-discriminatory manner.

Of particular note are the observations of Advocate General Wahl, who, in his opinion, expressed doubts as to whether the scientific evidence unequivocally supported the thesis that “non-ritual slaughter with stunning” is categorically less painful for the animal [28].

Turning to the issue of consumer transparency in food labeling, the 2019 decision in Oeuvre d’assistance aux bêtes d’abattoirs (OABA) v. Ministre de l’Agriculture et de l’Alimentation and Others is instructive. In that case, the Court addressed whether meat obtained via ritual slaughter—specifically halal—could be certified as organic [29]. The Court rejected this possibility, reasoning that slaughter conducted without prior stunning, even if compliant with Regulation (EC) No 1099/2009, could not guarantee the absence of animal suffering. Consequently, it was incompatible with the ethical standards underpinning the “organic” label.

According to the Court, the classification of meat as “organic” presupposes adherence to elevated animal welfare standards. Consumers, the Court argued, must be afforded clarity regarding the conditions under which animals were slaughtered. Therefore, denying organic status to meat derived from ritual slaughter served the legitimate aim of offering “certainty” to the end consumer. Only meat from animals that had been stunned prior to slaughter could rightfully bear the organic designation.

In his separate opinion, Advocate General Wahl acknowledged the legitimacy of this consumer-protective rationale but raised important counterpoints. Specifically, he noted that neither the relevant EU legislation nor the definitions governing organic certification explicitly exclude ritual slaughter. This omission, he suggested, may not represent a legislative gap but rather a deliberate choice by the EU legislator not to exclude halal or kosher products from organic classification [30].

In 2020, the Court revisited these issues in the landmark ruling Centraal Israëlitisch Consistorie van België and Others v. Vlaamse Regering [31]. The plaintiffs—representing Jewish and Muslim communities—contested Belgian legislation mandating pre-slaughter stunning, including a requirement for reversible electronarcosis in cases of ritual slaughter, as prescribed by a Flemish regional decree dated 7 July 2017. They argued that such provisions infringed their rights under Articles 10 and 22 of the Charter of Fundamental Rights of the European Union, which enshrine religious freedom and the principles of equality and non-discrimination.

The Court, however, upheld the legality of the regional measure. It reaffirmed the prerogative of Member States to impose restrictions on ritual slaughter where such measures are justified by public interests—such as animal welfare, public health, or morality—and are proportionate and non-discriminatory. Because the Flemish regulation did not target religious practice per se, but rather introduced a general requirement applicable to all slaughter methods, the Court found no violation of religious freedom.

The underlying hygiene and safety rationale behind the Flemish legislation—namely the restriction of ritual slaughter to specially licensed facilities—was also deemed legitimate. Nonetheless, this raises a deeper cultural consideration: the symbolic and ethical value that food prepared in accordance with religious traditions holds for Muslim communities. In Islamic thought, the act of slaughter is not merely functional but deeply symbolic, grounded in the ethical conviction that the animal’s life holds intrinsic value, thus justifying its consumption only under strict moral guidelines.

Ultimately, the Court’s ruling aligns with a “secular” European legal tradition, wherein sanitary standards, animal welfare, and public morality are prioritized. However, it appears to afford less weight to religiously informed ethical considerations around consumption. Viewed in this light, the judgment signals a further, incremental restriction on the permissibility of ritual slaughter without prior stunning—an outcome grounded in an increasingly robust interpretation of animal welfare.

By progressively affirming such jurisprudence, the Court appears to be implicitly recognizing a form of animal rights at the moment of slaughter, where moral, scientific, and economic rationales converge.

## 4. Discussion

The jurisprudential trajectory concerning ritual slaughter navigates a delicate equilibrium between the protection of religious freedom and the imperative of animal welfare. The imposition of restrictions on the slaughter of animals without prior stunning—enacted in jurisdictions such as Belgium, Denmark, and Sweden—has generated considerable controversy within both Islamic and Jewish communities. Within the European Union, the regulation of animal slaughter is governed by detailed normative frameworks designed to ensure minimal suffering and optimal welfare [20].

Given that animals are legally recognized as “sentient beings,” the practice of stunning prior to slaughter serves the purpose of mitigating the induction of adverse psychological states, most notably fear. This foundational principle is codified in Article 4 of the relevant regulation, which, albeit allowing for ritual slaughter, nonetheless affirms the primacy of animal welfare [18].

Religious traditions that prescribe ritual slaughter assert that the animal must approach the moment of death in a state of complete physical and psychological unalteration in order to maintain ritual purity and compliance with doctrinal requirements.

Although the European Convention on Human Rights (ECHR) guarantees the exercise of religious belief, it simultaneously imposes a dual limitation: the necessity of safeguarding public order and ensuring the protection of animal welfare [32].

In adjudicating such matters, the judiciary is called upon to conduct a meticulous assessment of necessity, suitability, and proportionality. Courts frequently invoke scientific evidence in their reasoning, which is considered inherently objective and rational: “in the argumentative phase, to scientific data which, by its nature, should stand as a solid element, since it is rational” [4].

Crucially, what emerges from the present analysis is “the twofold problem on the perspective of the right to life and humane treatment of an animal.” While the concept of a “right to life” for animals remains, in legal terms, a “non-right” or “impossible right,” the discourse is increasingly centered on humane treatment [33]. The degree of human moral advancement may well be measured by the consideration afforded to non-human life. Were the “right to life” to be affirmed in relation to animals, the ramifications for the food industry would be immediate and profound [34]. Consequently, legal discourse often pivots toward the notion of “animal welfare,” encompassing the moral issue of “animal suffering” [35] as well as transparency in meat labeling practices [36].

In assessing animal welfare standards, the European Union (EU), the Council of Europe (CoE), the Court of Justice of the European Union (CJEU), and individual Member States predominantly rely upon scientific data concerning physical pain and suffering associated with both conventional and ritual slaughter practices. However, a lack of scientific consensus persists, particularly regarding the critical temporal threshold that marks the final moments of an animal’s life—a threshold that may not align with prevailing human conceptions of “welfare.”

Going forward, it is anticipated that jurisprudence and legislative reforms will further refine the demarcation between constitutionally protected religious rites and evolving norms of animal welfare. This dynamic underscores the necessity for continuous legal, ethical, and scientific monitoring. In this light, the development of harmonized European guidelines appears imperative—guidelines capable of reconciling fundamental rights with the ethical imperatives of animal welfare, while curbing normative fragmentation across Member States.

## 5. Conclusions

The jurisprudence of the European Court of Justice—particularly in relation to ritual slaughter—possesses significant potential to influence national courts, including those in Italy. Pursuant to the principle of the hierarchy of legal sources, EU regulations and CJEU rulings hold primacy over conflicting national legislation. Accordingly, in resolving domestic legal controversies, national courts may formulate their own legal reasoning through interpretative engagement with EU law. Moreover, national judiciaries retain the prerogative to submit preliminary references to the CJEU on matters pertaining to the interpretation and validity of European law.

Yet, beyond the interpretative function of the judiciary, the broader legal landscape demands a sustained and interdisciplinary dialogue—one that includes jurists, theologians, ethicists, and animal welfare scientists. Such inclusive discourse is vital to ensuring that future regulatory frameworks are not only legally robust but also ethically attuned, capable of fostering mutual understanding across diverse cultural and religious milieus.

## Figures and Tables

**Table 1:** Key European case law on ritual slaughter in the context of religious freedom and animal welfare.

Case	Tribunal and Date of Final Decision	Why Is Important?
Cha’are Shalom Ve Tsedek v. France Application n. 27417/95	European Court of Human Rights (ECtHR), 27 June 2000	ECtHR acknowledged that ritual slaughter falls within the scope of religious freedom protected by Article 9 of the European Convention on Human Rights. However, it held that France’s refusal to authorize a second Jewish association for ritual slaughter did not violate the Convention, as members could still access meat compliant with their beliefs via existing channels.
Liga van Moskeeën en Islamitische Organisaties Provincie Antwerpen, VZW and others c. Vlaams Gewest Application n. C-426/16	Court of Justice of the European Union (CJEU), 29 May 2018	Restricting ritual slaughter to authorized slaughterhouses only does not directly violate religious freedom. Judges considered the increased costs and logistical burdens as indirect and acceptable.
Oeuvre d’assistance aux bêtes d’abattoirs (OABA) v. Minister of Agriculture and Food and Others Application n. C-497/17	Court of Justice of the European Union (CJEU), 26 February 2019	Meat from animals slaughtered without prior stunning cannot be labelled as organic. Stunning is essential to meet the highest animal welfare standards required for organic certification. There is a legitimate expectation on the part of consumers who see the organic label and believe the animal was slaughtered in a compassionate manner.
Centraal Israëlitisch Consistorie van België and Others v. Flemish Government Application n. C-336/19	Court of Justice of the European Union (CJEU), 17 December 2020	Requiring reversible stunning during ritual slaughter is lawful under EU rules. It balances animal welfare concerns with religious freedom without disproportionately restricting the latter.
Executief van de Moslims van België and Others v. Belgium Application n. 16760/22 and 2107/23	European Court of Human Rights (ECtHR), 13 February 2024	The ECtHR held that Belgium’s ban on ritual slaughter without prior stunning does not violate the European Convention on Human Rights. The Court found the measure justified under the protection of public morals, acknowledging animal welfare as a legitimate aim.

## Data Availability

No new data were created or analyzed in this study. Data sharing is not applicable to this article.
